# Setting thresholds to determine COVID-19 activity levels using the mean standard deviation (MSD) method, England, 2022–2024

**DOI:** 10.2807/1560-7917.ES.2024.29.45.2400696

**Published:** 2024-11-07

**Authors:** Mary A Sinnathamby, Tania Bourouphael, Jacob Boateng, Magali Collonnaz, Catherine Quinot, Nurin Abdul Aziz, Suzanne Elgohari, Rebecca E Green, Gavin Dabrera, Jamie Lopez-Bernal, Alex Allen

**Affiliations:** 1 Respiratory Virus Section, Immunisation and Vaccine-Preventable Diseases Division, Public Health Programmes, United Kingdom Health Security Agency (UKHSA), London, United Kingdom

**Keywords:** COVID-19, thresholds, epidemiology, influenza

## Abstract

We developed a new activity level setting threshold method, the mean standard deviation (MSD) method to quantify COVID-19 activity levels. This has been validated against the moving epidemic method (MEM), which has been used for influenza for many years, and we observed very similar results. The MSD method can prove to be a tool to use for respiratory viruses with limited historical data or seasonality to quantify activity levels when other respiratory viruses are also circulating.

Since its emergence in 2020, the severe acute respiratory syndrome coronavirus 2 (SARS-CoV-2), the causative agent of COVID-19, has been circulating worldwide to date with increases in circulation noted as new variants emerge [1,2]. Testing regimes have changed across many countries as the World Health Organization (WHO) announced the COVID-19 pandemic to no longer be a public health emergency of international concern (PHEIC) in May 2023 [3]. As such, to be able to monitor and interpret activity, i.e. virus circulation levels during the winter period across three respiratory viruses influenza, SARS-CoV-2 and respiratory syncytial virus (RSV) in a consistent and somewhat similar manner, we have developed and validated a new threshold setting method, the mean standard deviation (MSD) to set activity thresholds for COVID-19 surveillance systems in England [4].

## Rationale

In England, since 1 April 2023, COVID-19 testing is mainly done in symptomatic adults and children within hospital settings for clinical management [5]. Given these changes and the circulation of SARS-CoV-2 not observing a strict seasonality, with multiple waves of circulation seen per year, and substantial baseline activity, compared with other respiratory viruses like influenza, it has been difficult to quantify its activity levels [6]. Traditionally for influenza surveillance systems, activity thresholds have been set following the WHO Pandemic Influenza Severity Assessment (PISA) guidelines using the moving epidemic method (MEM) to monitor activity levels season on season, however, this requires at least five seasons’ worth of consistent historical data [4,7]. While MEM thresholds can be set for influenza and RSV data due to the availability of historical and consistent data, this is not possible for COVID-19 due to the recency of the virus and the lack of a defined seasonal pattern to date, given the frequency in viral evolution (i.e. variants), changes in testing regimes and waning immunity to infection in the population following vaccination/campaigns.

## Setting thresholds

We collated COVID-19 data from four main surveillance systems: (i) primary care (Royal College of General Practitioners Research and Surveillance Centre (RCGP RSC)) swabbing scheme, (ii) laboratory-based testing surveillance through the second generation surveillance system (SGSS), (iii) hospitalisations to all levels of care including intensive care unit (ICU)/ high dependency unit (HDU) and (iv) ICU/HDU admissions, both through the severe acute respiratory infection (SARI)-Watch surveillance system [8].

We analysed data for each surveillance system from week 27 2022 to 26 2024 (i.e. 4 July 2022–30 June 2024), taking each 52 weeks as one season, to evaluate trends in waves of activity.

**Figure 1:**
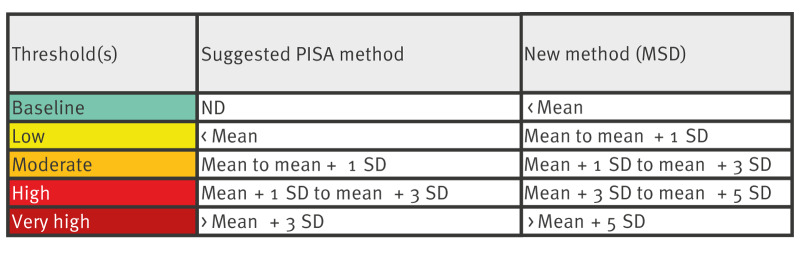
Proposed thresholds for monitoring of COVID-19 in the population

MSD: mean standard deviation; ND: not determined; PISA: Pandemic Influenza Severity Assessment; SD: standard deviation.

We first assessed the mean and standard deviation (SD) across the proposed time period using the suggested PISA method for threshold bands as taken from the latest PISA guidance [4] (Figure 1). Upon review, the suggested PISA method did not allow for a baseline activity threshold which may be interpreted as activity to be consistently above expected levels. Additionally, the use of a baseline threshold would align with surveillance systems for influenza and RSV which use the five threshold bands of the MEM method. Given this, we decided to adapt the suggested PISA method to a new method which calculates five threshold bands (baseline, low, moderate, high and very high) to monitor COVID-19 activity levels across surveillance systems (Figure 1).

**Figure 2:**
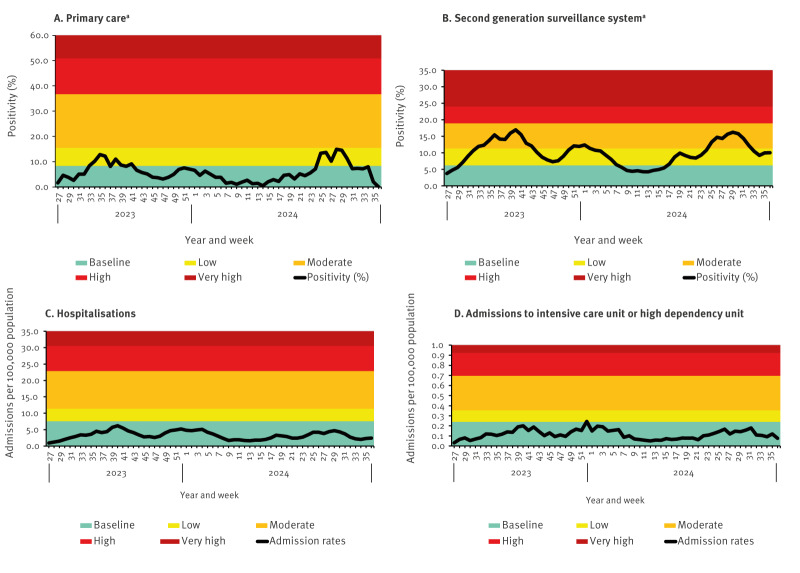
Data on COVID-19 surveillance with thresholds set by mean standard deviation method, England, week 27 2023–week 36 2024

^a^ Sampling within the primary care (Royal College of General Practitioners Research and Surveillance Centre (RCGP RSC)) swabbing scheme.

The adaptation of the high and very high thresholds to use 3 SD and 5 SD was determined by evaluating the distribution of these bands across the four surveillance systems’ data. Upon testing the use of 4 SD and 6 SD, we noted that the threshold cut-offs were either too wide or too narrow so activity levels would fluctuate often and not provide a consistent and accurate picture of activity in general if activity were to reach these threshold cut-offs (Figure 2).

Thresholds based on data from week 27 2022 to week 26 2023 were used, 4 SD and 6 SD were used for high and very high thresholds.

## Setting the time period

To take into account the post-PHEIC/1 April 2023 testing changes and reflect activity levels more accurately, the use of data from one season to calculate thresholds was decided for the upcoming season, 2024/25.

**Figure 3:**
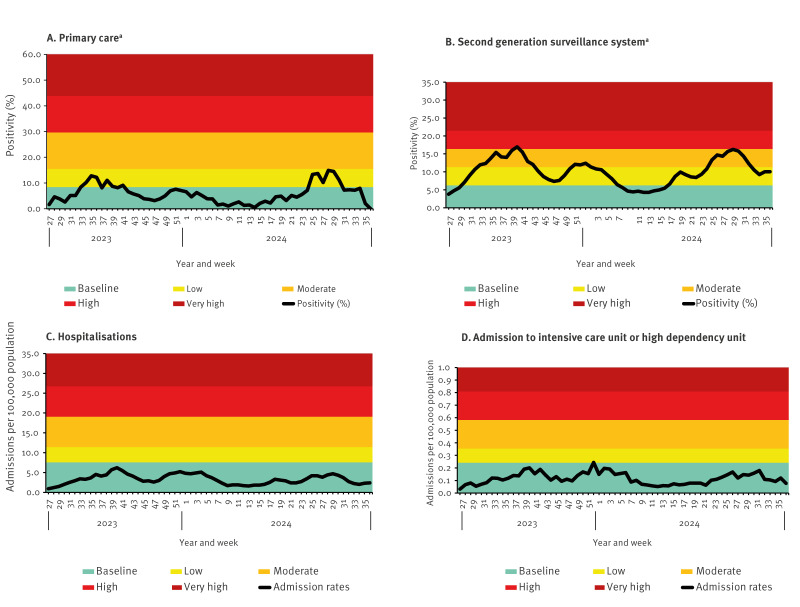
Data on COVID-19 surveillance with thresholds set by the finalised defined mean standard deviation method, England, week 27 2023–week 36 2024

^a^ Sampling within the primary care (Royal College of General Practitioners Research and Surveillance Centre (RCGP RSC)) swabbing scheme.

Subsequently, the application of data from one season (week 27 2022–week 26 2023) was adopted and applied to the data of 2023/24 season (week 27 2023–week 26 2024) which proved to be in line with activity levels observed during the season. For example, hospitalisations and ICU/HDU admissions were indeed very low in comparison to previous years and therefore not expected to have breached the baseline threshold (Figure 3).

Thresholds based on data from week 27 2022 to week 26 2023 were used.

## Validating the new method

**Figure 4:**
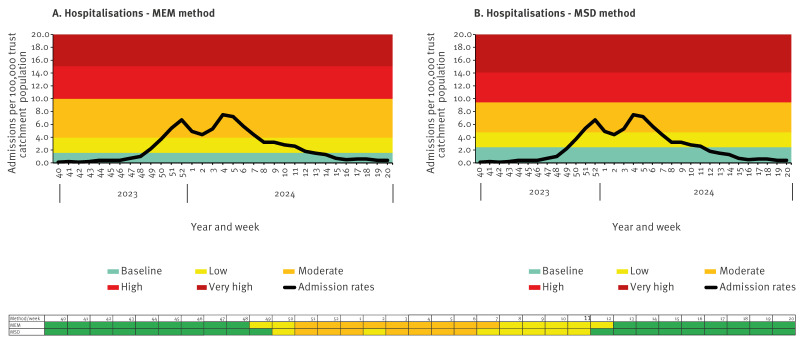
Validation of the moving epidemic method against a new mean standard deviation method using influenza data from severe acute respiratory infection surveillance (SARI-Watch) for hospital admissions, England, week 40 2023–week 20 2024

MEM: moving epidemic method; MSD: mean standard deviation.

**Figure 5:**
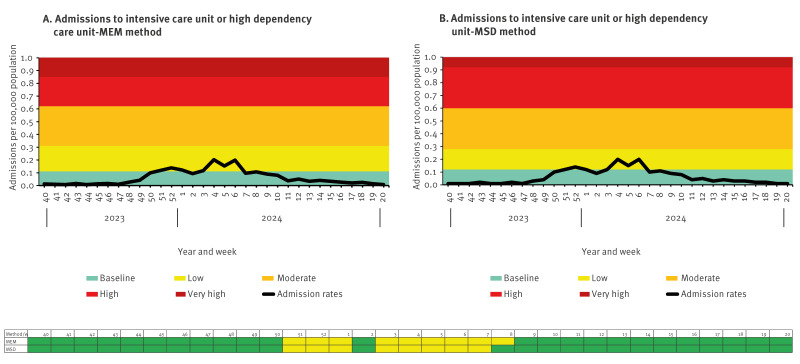
Validation of the moving epidemic method against a new mean standard deviation method using influenza data from severe acute respiratory infection surveillance (SARI-Watch) for intensive care unit or high dependency unit admissions, England, week 40 2023–week 20 2024

MEM: moving epidemic method; MSD: mean standard deviation.

To validate the new method, we used the SARI-Watch surveillance systems which also collate influenza hospitalisation (all levels of care including ICU/HDU) and ICU/HDU admission data to compare activity levels produced in comparison to its traditionally used MEM threshold method. We used 2023/24 data (week 40 2023–week 20 2024) to follow a usual influenza season with MEM thresholds modelled based on data from seasons 2015/16, 2016/17, 2017/18, 2018/19 and 2022/23 (excluding the pandemic seasons) (Figure 4, 5).

Thresholds based on data from seasons 2015/16, 2016/17, 2017/18, 2018/19, 2022/23 were used.

Thresholds based on data from seasons 2015/16, 2016/17, 2017/18, 2018/19, 2022/23 were used.

This analysis highlighted that despite only using influenza data from one season, the new threshold matched very closely to those produced by the MEM method (which uses data from five seasons) for both hospitalisation and ICU/HDU admissions of influenza (Figure 4, 5).

## Discussion

Integrated surveillance for respiratory viruses, particularly for influenza and COVID-19, has been advocated for, particularly in Europe, since 2021 [9]. It has growingly also become important to be able to differentiate activity levels between respiratory viruses to aid with public health communication particularly during the winter months. The WHO PISA guidance suggests methods of threshold setting to monitor activity levels for influenza surveillance which have been adopted for several seasons (pre and post the COVID-19 pandemic) [4,6]. We have adapted suggested thresholds to create a new threshold setting method (MSD) which fits COVID-19 activity trends in England. Moreover, we have validated this new method using influenza data from a long-standing surveillance system to compare activity levels produced by the established existing method (MEM) and our new method (MSD) which showed very similar activity thresholds and levels. This is indicative of the potential robustness and appropriateness of our new method for use with COVID-19 data.

To our knowledge, this is the first study attempting to set activity thresholds for the surveillance of COVID-19 since its emergence. It is important to note that due to the lack of seasonality and baseline activity levels COVID-19 observes, this method may be unstable until enough data have been accumulated to reflect current activity. Therefore, it is important to monitor or review its ability to predict activity thresholds accurately through careful review, as we will do for the upcoming 2024/25 season (where data from one year are used) and if required adapted year on year. Therefore, it is also important to note that it is recommended that this method can be used until we accumulate historical data to ensure appropriate thresholds are set yearly until we have enough data (5 years) to apply the MEM method. Our analysis reflected the 2023/24 COVID-19 season activity well. However, it is important to note that for hospitalisations and ICU-HDU, the rates in 2022/23 were considerably higher for most of the year and, therefore, the 2023/24 baseline was high.

## Conclusion

Our new method to set activity thresholds for the surveillance of COVID-19 could also be applied as a potential interim method for other respiratory viruses, which do not show seasonality, until enough consistent historical data are available to use other methods such as the MEM. As countries in the northern hemisphere enter the 2024/25 winter season, the MSD method could aid in the interpretation and communication of not only COVID-19 activity itself, but also its comparison to other circulating respiratory viruses such as influenza.
